# Male Sexual Victimization by Women: Incidence Rates, Mental Health, and Conformity to Gender Norms in a Sample of British Men

**DOI:** 10.1007/s10508-023-02717-0

**Published:** 2023-10-18

**Authors:** Jasmine Madjlessi, Steve Loughnan

**Affiliations:** 1 Department of Philosophy, Psychology, and Language Sciences, University of Edinburgh, Edinburgh, UK; 2 Faculty of Law and Criminology, Ghent University, Ghent, 9000 Belgium

**Keywords:** Sexual victimization, Male victims, Female perpetration, Gender norms, Mental disorders

## Abstract

Male sexual victimization by women is often neglected within psychological research (Fisher & Pina, 2013). Not only is the topic understudied, incidence rates and associated psychological impacts are inconsistent across the literature (Depraetere et al., 2020; Peterson et al., 2011). The present study provides an additional estimate of male sexual victimization by women, explores its association with victim mental disorders, and examines the potential moderating role of conformity to gender norms. A sample of 1124 heterosexual British men completed an online survey consisting of a modified CDC National Intimate Partner and Sexual Violence Survey, and measures of anxiety, depression, post-traumatic stress disorder, and conformity to masculine norms. In the present sample, 71% of men experienced some form of sexual victimization by a woman at least once during their lifetime. Sexual victimization was significantly associated with anxiety, depression, and post-traumatic stress disorder. However, conformity to masculine gender norms was not a significant moderator between victimization and mental disorders. These findings further illuminate the occurrence of male sexual victimization by women, as well as the importance of continued research on the topic.

## Introduction

Male sexual victimization by women is a topic often misunderstood by the public, understudied by researchers, and overlooked by public policy (Fisher & Pina, 2013; Peterson et al., 2011; Weiss, 2010). The incidence and psychological implications of sexual victimization, such as anxiety, depression, and post-traumatic stress disorder (PTSD), are well evidenced in studies of women (Campbell et al., 2009; Koss, 1993a; Resick, 1993). However, there is far less research on sexual victimization in male samples, particularly regarding female perpetrators (Fisher & Pina, 2013), and its consequences for men’s mental health. Within the small body of literature on male sexual victimization, reports of the incidence rate and associated mental disorders are conflicting (Peterson et al., 2011). This may be due in part to prevailing gender norms that impact whether victims report incidents of victimization and associated mental disorders.

The first aim of the present study was to determine a lifetime incidence rate of male sexual victimization by women in a sample of British men. The second aim was to explore whether male sexual victimization by women—defined by the incidence of victimization, types of victimization experienced, and number of incidents—is linked to subsequent mental disorders, namely generalized anxiety, depressive disorder, and PTSD. The final aim was to examine whether conformity to masculine gender norms was a moderating factor in the potential relationship between male sexual victimization by women and mental disorders.

A general definition of sexual victimization is the experience of any unwanted sexual activity committed or attempted through physical force, psychological coercion, or the exploitation of an inability to consent (Basile & Saltzman, 2002; Spangaro et al., 2013). The widespread occurrence and devastating impacts of female sexual victimization are well-established (Koss, 1993a; Resick, 1993). Sexual victimization increases the likelihood of negative health outcomes across a range of domains; female victims may suffer from physical symptoms, developmental deficits, social difficulties, sexual dysfunction, and neurological impairment (e.g., Kuwert et al, 2014; Mash & Wolfe, 2016; McCauley et al., 1997; Wilson et al., 2011**)**. In addition, female victims face an increased risk of mental disorders, such as anxiety, depression, PTSD, eating disorders, substance abuse disorders, and suicidality (Campbell et al., 2009; Chen et al., 2010). A qualitative review by Campbell et al. reported that up to 40% of female victims experience generalized anxiety and up to 51% of female victims meet the diagnostic criteria for depression. Female victims may also experience distressing memories and dreams, flashbacks, hypervigilance, feelings of shame and fear, and an inability to experience happiness due to exposure to sexual trauma (American Psychiatric Association [APA], 2013). Accordingly, it is unsurprising that some of the highest PTSD rates in the population are among sexual victims (APA, 2013), with up to 65% of female victims experiencing PTSD (Campbell et al., 2009). Undoubtedly, sexual victimization is worthy of study due to its association with subsequent psychological suffering and impaired functioning in important areas of daily life.

### Incidence Rates of Male Victimization

It is reasonable to expect that male victims may experience similar levels of mental disorders as well. Yet, male sexual victimization is far less studied (Fisher & Pina, 2013), particularly so with regards to female perpetrators (Stemple et al., 2017). The occurrence of male sexual victimization and the psychological distress male victims may suffer has not only received considerably less scientific attention, but results are inconsistent across studies (Krug et al., 2002; Peterson et al., 2011). The incidence rates of male sexual victimization range widely, from less than one percent to 73%, with the highest reported estimate of specifically female-perpetrated victimization at 70% (Depraetere et al., 2020; Peterson et al., 2011). This disparity in incidence rates is likely due to differences in research methodologies (Depraetere et al., 2020; Peterson et al., 2011). Results of incidence studies may differ depending on the research criterion used, such as the age victimization occurred (e.g., childhood, adulthood, lifetime), the gender of the perpetrator, the phrasing of questions, and operationalized definition of sexual victimization. Notably, studies that report higher estimates of victimization tend to use behaviorally specific questions that leave little room for participant interpretation (Koss, 1993b). These studies also use broader definitions of sexual victimization that include less severe forms (e.g., harassment, unwanted kissing, groping), male-specific forms of victimization (e.g., being made to penetrate), and different coercive tactics beyond physical force (e.g., verbal pressuring, drugging; Depraetere et al., 2020; Peterson et al., 2011). In short, the current literature on male victimization is unclear and methodological differences might partially account for this diversity of findings.

### Impacts of Male Sexual Victimization on Mental Health

Beyond incidence estimates, a greater understanding of the association between male sexual victimization and mental disorders is needed, with an unclear picture emerging across existing literature. Many studies including male victims support that sexual victimization is associated with a range of mental disorders. For example, Peterson et al. (2011) conducted a review of 10 studies comparing men who experienced adulthood sexual victimization with non-victimized counterparts. All 10 studies indicated that victimized men experienced more mental disorders, including anxiety, depression, alcohol abuse, and suicidality, compared to men who had not been victimized. Further, multiple studies report that there is no gender difference in mental disorders related to sexual victimization. A meta-analysis by Chen et al. (2010) indicated that lifetime sexual victimization is associated with a range of mental disorders, such as anxiety, depression, PTSD, eating disorders, sleep disorders, substance abuse, and suicide attempts, regardless of the victim’s gender. Similarly, a meta-analytic review of sexual victimization by Dworkin et al. (2017) supported that victims exhibit significantly more symptoms of anxiety (g = 0.53), depression (g = 0.60), and trauma- and stressor-related conditions such as PTSD (g = 0.71), with no pathological differences based on gender. The results of multiple meta-analyses provide convincing evidence that the victim’s gender does not differ the likelihood of experiencing mental disorders related to sexual victimization.

Yet, it is helpful to examine the studies that report gender differences in mental disorders related to sexual victimization. Multiple studies indicate that men report less negative psychological impacts following sexual victimization. Studies with college student aged male victims found that they report less short- and long-term psychological impact compared to female victims (e.g., O'Sullivan et al., 1998; Struckman-Johnson, 1988). One such study revealed that male victims were less likely to report feeling bad (27% vs. 88%) and more likely to report feeling neutral (46% vs. 12%) or good (27% vs. 0%) shortly after an unwanted sexual experience, compared to female victims (Struckman-Johnson, 1988). These self-reported results by male victims of little to no psychological distress following sexual victimization are also prevalent in studies of male sexual victimization by women (Krahé et al., 2003). For example, college aged men reported that sexual aggression by women experienced since the age of 16 was more likely to leave no impact (47% vs. 21%) and less likely to leave a moderate to severe impact (23% vs. 47%) compared to sexual aggression perpetrated by men (Struckman-Johnson & Struckman-Johnson, 1994). Notably, these studies rely on self-reported data in which psychological impact is not clearly defined and measured by the researchers but left for the participants to interpret and evaluate for themselves. Thus, it is difficult to determine whether the results reflect a lack of psychological impact or the reluctance for male victims to acknowledge and report emotional distress.

By contrast, a few studies indicate that male sexual victimization is related to higher levels of mental disorder symptomology (e.g., Elliott et al., 2004; Kimerling et al., 2002; Struckman-Johnson & Struckman-Johnson, 2006). A large sample study (*n* = 941) on adulthood sexual victimization found that assaulted men were more likely than assaulted women to report greater trauma-related symptoms, which persisted over time (Elliott et al., 2004). Another study of medical charts at a rape treatment center indicated that male victims had significantly higher rates of current psychological symptoms (41% vs. 11%), as well as lifetime history of psychological disorders (55% vs. 29%) and psychological hospitalizations (52% vs. 18%) compared to female victims (Kimerling et al., 2002). The authors postulate that it is possible that men may experience greater psychological distress compared to women following sexual victimization, particularly if male victims struggle with feelings of a compromised masculine identity (Kimerling et al., 2002; Peterson et al., 2011).

### The Influence of Gender Norms

These studies are indicative of inconsistent findings across the literature, yet the results may provide unique insights into the multiple facets of male sexual victimization. The conflicting results across studies may reflect the role of socialized gender norms and fears about maintaining masculine identity. Gender is not based on biological differences but is defined by cultural notions of appropriate masculine and feminine behavior and characteristics (Ferris & Stein, 2014; Weiss, 2010). Gender norms, which refer to the socially constructed cultural expectations for how men and women should behave and interact with each other, may impact how victims report and think about sexual victimization (Depraetere et al., 2020). Norms of masculinity are counter to social constructions of sexual victimhood (Weiss, 2010). There is a cultural myth that male sexual victimization by women is not possible, either through physical force or through psychological coercion, because men are supposed to be physically dominant and aggressive, independent, and able to protect themselves, whereas women are supposed to be the opposite: gentle, submissive, and weak (Ferris & Stein, 2014; Kite, 2001; Myers, 2013; Prentice & Carranze, 2002). Thus, it is important to study male sexual victimization by women, not only due to the deficiency of existing literature, but because of the unique implications of female perpetration on mental health and threats to gender identity in male victims.

Notions of gender extend to norms of sexuality as well (Ferris & Stein, 2014). Sexuality is also a social construct, in which appropriate attitudes and behaviors around sex are shaped by cultural norms (Weiss, 2010). Men are expected to behave as sexual opportunists, in which they initiate and pursue sexual opportunities with women, who are expected to be sexual gatekeepers (Abdullah-Khan, 2008; Gupta, 2000; O’Sullivan et al., 1998; see Anderson et al., 2021). Based on social norms, sex with a woman should always be a desirable outcome for men; thus, those who acknowledge sex with a woman was unwanted or forced counter norms of traditional masculinity and sexual scripts. Social norms also indicate that men should not experience or express emotional suffering, as it conflicts with masculine norms of stoicism and strength (Weiss, 2010). Men who appear to deviate from their prescribed social roles may experience stigma and feel that they must defend their masculinity, as gender norms for men may be more rigidly defined than norms for women (Ferris & Stein, 2014; Kite, 2001). Thus, male victims may seek to deny victimization and the severity of their experiences in order to preserve their sense of masculine identity. Conversely, male victims may experience a unique dimension of emotional suffering due to threats to their sense of self and questions to their masculine identity posed by sexual victimization. In essence, although there is convincing evidence that sexual victimization is associated with numerous mental disorders, regardless of victim gender, it is possible that gender norms moderate the relationship between sexual victimization and mental disorders.

### The Current Study

Sexual victimization is undoubtably important to study, especially due to the potential psychological suffering it may cause. Yet, the occurrence and effects of male sexual victimization are under-researched, particularly regarding female perpetrators. The present study aimed to provide an estimate of male sexual victimization by women, to explore mental disorders associated with victimization, and to analyze gender norms as a potential moderating factor in the relationship between sexual victimization and mental disorders. Previous incidence estimates have ranged widely from < 1% to 73% (Depraetere et al., 2020; Peterson et al., 2011). Despite relatively convincing evidence pointing toward a significant relationship between sexual victimization and psychological suffering irrespective of gender, there are conflicting studies indicating that male victims may be either less likely to experience psychological distress following sexual victimization or, inversely, may be more prone to developing mental disorders. It is possible that male sexual victimization is moderated by conformity to masculine gender norms, which could affect how male victims perceive their own victimization and experience psychological distress. The moderation was conceptualized so that higher conformity to masculine gender norms would have some impact on rates of mental disorders, either through higher or lower mental disorder symptomology. Compared to previous research, the present study utilizes a large sample of British men, deploys a robust measure of sexual victimization, and uses three nuanced victimization variables. However, no a-priori hypotheses were made due to conflicting evidence across the literature.

## Method

### Participants

A total of 1190 adults from the United Kingdom participated in the online study in exchange for payment of £1 through *Prolific Academic.* The purpose of the questionnaire was advertised as a study on “men’s sexual experiences and mental health.” The description forewarned that questions would be asked about non-consensual sexual experiences. Data were collected from 512 participants in late May 2022 and 678 participants in early June 2022. Participants were pre-screened through *Prolific’s* internal filters to be heterosexual British men. Age, gender, and sexuality were the only demographic information collected. Further demographics were not gathered with the aim that greater anonymity may increase participant willingness and accurate reporting (Rosenbaum & Langhinrichsen-Rohling, 2006). Twenty-two participants indicated that they did not identify as heterosexual and 26 participants indicated that they did not identify as male; these participants were excluded from data analysis. Eighteen participants, being 0.016% of the sample, completed less than 100% of the survey and were also excluded from analysis. This deviated from the preregistration which stated an inclusion criterion of 90% completion. The final sample consisted of 1,124 heterosexual adult males from the United Kingdom between the ages of 18 and 84 (*M* = 42; *SD* = 13.57). A power analysis using G*POWER was conducted prior to participant recruitment to ensure the initial sample of 320 participants exceeded that necessary to meet 60% power. A post-hoc power analysis suggested that the final sample (*n* = 1124) achieved 100% power (*a* = 0.05, two-tailed). The exclusion criterion and statistical approach were pre-registered (https://osf.io/d672x/).

### Procedure

The survey was administered online using *Qualtrics.* A sexual victimization questionnaire was presented first, out of consideration for potential emotional burnout. Subsequently, participants were presented with the remaining measures on conformity to gender norms, PTSD, anxiety, and depression in random order. At the conclusion of the study, participants were debriefed and offered mental health and male sexual victimization resources.

### Measures

All measures are available online (https://osf.io/d672x/).

#### Sexual Victimization

Sexual victimization was measured using a modified version of the CDC’s National Intimate Partner and Sexual Violence Survey (NISVS; Black et al., 2011). The original survey was created to collect national data through telephone interviews on both male and female experiences with physical, sexual, and psychological abuse. The format of the questionnaire was modified to fit an online survey for the current study (*a* = 0.89). The modified survey included 25 questions regarding sexual victimization, as well as two questions regarding control of reproductive health that were not used in the analysis, as they were beyond the scope of the current study.

The phrasing of the original questions was modified to be specific to male victims and female perpetrators. For example, the original survey question of “How many people have ever kissed you in a sexual way? Remember, we are only asking about things that you didn’t want to happen” was changed to “How many times has a woman kissed you in a sexual way? Remember, I am only asking about things that you didn’t want to happen” (Black et al., 2011). The survey was also modified to include questions about experiences with unwanted object penetration, digital/manual stimulation, and, where appropriate, the term “penetrate” was changed to “touch” to reflect a wider range of experiences. Participants were given four multiple-choice options to respond to the questions (i.e., “Never,” “Once,” “Twice,” and “More than twice”).

To obtain a more nuanced understanding of victimization, three variables were created to examine the incidence, breadth, and depth of unwanted sexual experiences. For the first victimization variable, incidence of victimization, responses to the modified NISVS survey were dichotomized so that 0 reflected no affirmative answers and 1 reflected affirmative answers to at least one item on the scale. Thus, participant scores ranged between 0 and 1. The second victimization variable, breadth of victimization, reflected the types of sexual victimization experienced across 25 forms of sexual victimization. Thus, participant scores ranged from 0 to 25. The third victimization variable, depth of victimization, reflected how many times sexual victimization occurred across 25 forms of abuse. Thus, participant scores ranged from 0 to 75.

#### Conformity to Masculine Gender Norms

The short form Conformity to Masculine Norms Inventory (CMNI-30; Levant et al., 2020) was used to assess conformity to masculine norms (*a* = 0.83). Across 30 items, participants were tested on ten sub-factors of masculine norms (i.e., emotional control, winning, playboy, violence, heterosexual self-presentation, pursuit of status, primacy of work, power over women, self-reliance, risk-taking) with three respective questions for each sub-factor. Participants answered on a 6-point Likert scale ranging from 0 (“strongly disagree”) to 5 (“strongly agree”).

#### Mental Disorders

##### Anxiety

The GAD-7 (Spitzer et al., 2006) was used to measure generalized anxiety disorder based on the DSM-IV criteria (*a* = 0.93). Participants were presented with seven items, to which they could respond through a four-point Likert scale from 0 (“not at all”) to 3 (“nearly every day”). For example, “Over the last 2 weeks, how often have you been bothered by little interest or pleasure in doing things?”

##### Depression

The PHQ-9 (Kroenke et al., 2001) was used to measure depression based on the DSM-IV criteria (*a* = 0.91). Participants were presented with nine items, to which they could respond through a four-point Likert scale from 0 (“not at all”) to 3 (“nearly every day”). For example, “Over the last 2 weeks, how often have you been bothered by not being able to stop or control worrying?”

##### Post-Traumatic Stress Disorder

The PTSD Checklist for DSM-5 (PCL-5; Weathers et al., 2013) was used to measure symptoms of post-traumatic stress disorder (*a* = 0.96). Participants were presented with 20 items, to which they could respond through a five-point Likert scale from 0 (“not at all”) to 4 (“extremely”). For example, “In the past month, how much were you bothered by repeated, disturbing, and unwanted memories of the stressful experience?”

### Statistical Procedure

The statistical program *R* was used to determine (1) the incidence rate of male sexual victimization by women, (2) whether victimization was associated with mental disorders, and (3) whether conformity to masculine norms moderates the potential relationship between victimization and mental disorders (R Core Team, 2023). The data were cleaned to ensure that categorical variables were appropriately encoded, reverse coding was conducted where necessary, and impossible values were transformed to *NA*. Cook’s Distance tests were conducted, revealing no influential observations to affect the outcome of analysis (*D*_i_ < 0.004), and thus, there was no outlier removal. Anxiety, depression, PTSD, and conformity to masculine norms were operationalized as *z*-scores. Victimization, the predictor variable, was operationalized by incidence, breadth, and depth of unwanted sexual experiences.

First, to estimate the incidence of male sexual victimization by women, responses to the modified NISVS survey were dichotomized, in which 0 reflected no reported victimization and 1 reflected reported victimization. The sum of 1’s was totaled and converted to a percentage. Second, to explore whether victimization was associated with mental disorders in the sample, linear regressions were conducted with victimization as the predictor and mental disorders as the outcome. To avoid issues of multi-collinearity, the three victimization variables were run in separate linear regression models, resulting in nine models. Finally, linear regressions were conducted with the interaction of conformity to masculine norms to determine the moderating effect on the relationship between victimization and mental disorders. Given that data from all variables (excluding conformity to masculine norms) violated the normality assumption of linear regression, a bootstrapping procedure was used based on 1000 bootstrap samples (Fox, 2016; Russell & Dean, 2000). Significance was determined at *p* < 0.001.

## Results

Pearson’s correlations and descriptive statistics were computed for all variables. Correlations presented in Table 1 show that variables were correlated in expected directions (e.g., mental disorder variables significantly correlate with each other). Results revealed that 71% of the sample reported experiencing male sexual victimization by women. In terms of frequency, 57% of the sample were victimized more than once and 45% of the sample experienced sexual victimization more than twice. Analysis of responses to individual questions indicated that 39.80% experienced attempted or completed forced vaginal/anal penetration (see Table 2). Further, the sample reported that sexual victimization occurred by force or threats of physical harm 4.77% of the time, by pressuring 33.00% of the time, and by exploitation of inebriation or the inability to consent 29.40% of the time. In short, we found considerable evidence of victimization.

**Table 1:** Descriptive statistics and correlations for study variables

Variable	*M* (*SD*)	*n* (Range)	Correlations						
			1	2	3	4	5	6	7
1. Depression	0.66 (0.64)	1,124 (0–3)	–						
2. Anxiety	0.68 (0.72)	1,124 (0–3)	.86*	–					
3. PTSD	0.70 (0.77)	1,124 (0–4)	.87*	.85*	–				
4. Masculine norms	1.85 (0.55)	1,124 (0.03–4.4)	.16*	.18*	.19*	–			
5. Incidence	0.71 (0.45)	1,124 (0–1)	.17*	.16*	.20*	.12*	–		
6. Breadth	3.50 (4.10)	1,124 (0–25)	.26*	.24*	.31*	.13*	.54*	–	
7. Depth	6.71 (9.31)	1,124 (0–70)	.26*	.24*	.30*	.13*	.46*	.94*	–

*p < .001. Absolute ranges are as follows: depression 0–3; anxiety 0–3; PTSD 0–4; masculine norms 0–5; incidence 0–1; breadth 0–25; depth 0–75

**Table 2:** Percentage of sample that experienced sexual victimization by category

Total	71%
Viewed exposure or masturbation	21%
Forced exposure	15%
Forced viewing or participation in sexual pictures or movies	9%
Public harassment	26%
Kissing	32%
Fondle or grabbing	45%
Drunk, high, passed out, or unable to consent	
Perform vaginal sex	15%
Perform anal sex	4%
Receive anal sex	4%
Perform oral sex	6%
Receive oral sex	13%
Receive digital stimulation	25%
Perform digital stimulation	11%
Physical force or threats of physical harm	
Perform vaginal sex	3%
Perform anal sex	1%
Receive anal sex	1%
Perform oral sex	2%
Receive oral sex	2%
Receive digital stimulation	2%
Perform digital stimulation	2%
Attempted but not completed	
Vaginal sex	36%
Oral or anal sex	25%
Pressured sexual activity	
Telling lies, making promises, threatening relationship, threaten rumors	19%
Wearing down by repeated requests, showing unhappiness	20%
Using authority	5%

Linear regressions were subsequently conducted to determine whether incidence, breadth, and depth of sexual victimization are associated with mental disorders. The results reveal that sexual victimization is statistically associated with anxiety, depression, and PTSD, controlling for participant age and gender norm conformity (see Tables 3, 4, 5).

**Table 3:** Hierarchical regressions for incidence of victimization

	Model 1: DV = Anxiety						Model 2: DV = Depression						Model 3: DV = PTSD					
	*B*	*SE*	95% CI		*β*	*R*_adj_^2^	*B*	*SE*	95% CI		*β*	*R*_adj_^2^	*B*	*SE*	95% CI		*β*	*R*_adj_^2^
			*LL*	*UL*					*LL*	*UL*					*LL*	*UL*		
Step 1						.05^***^						.05^***^						.04^***^
Constant	.67^***^	.09	.48	.84			.67^***^	.09	.50	.85			.66^***^	.09	.47	.84		
Age	−.02^***^	.00	−.02	−.01	−.22		−.02^***^	.00	−.02	−.01	−.22		−.02^***^	.00	−.02	−.01	−.21	
Step 2						.08^***^						.08^***^						.10^***^
Constant	.37^***^	.10	.19	.56			.36^***^	.10	.17	.55			.29^**^	.09	.12	.66		
Age	−.01^***^	.00	−.02	−.01	−.19		−.01^***^	.00	−.02	−.01	−.19		−.01^***^	.00	−.02	−.01	−.18	
Victimization	.29^***^	.06	.17	.41	.13		.32^***^	.06	.21	.44	.15		.38^***^	.06	.27	.48	.17	
Norm conformity	.13^***^	.03	.07	.19	.13		.11^***^	.03	.04	.18	.11		.14^***^	.03	.07	.20	.14	
Step 3						.08^***^						.08^***^						.09^***^
Constant	.38^***^	.10	.20	.58			.36^***^	.10	.17	.55			.28^**^	.10	.11	.48		
Age	−.01^***^	.00	−.02	−.01	−.19		−.01^***^	.00	−.02	−.01	−.19		−.01^***^	.00	−.02	−.01	−.18	
Victimization	.29^***^	.06	.17	.40	.13		.32^***^	.06	.19	.42	.14		.38^***^	.06	.26	.48	.17	
Norm conformity	.16^**^	.05	.06	.25	.16		.15^**^	.05	.05	.25	.15		.14^*^	.04	.06	.23	.14	
Vic × Norms	−.04	.06	−.16	.09	−.03		−.06	.07	−.19	.08	−.05		−.06	.06	−.12	−.10	.00	

^*^p < .05. ^**^p < .01. ^***^p < .001

**Table 4:** Hierarchical regressions for breadth of victimization

	Model 1: DV = Anxiety						Model 2: DV = Depression						Model 3: DV = PTSD					
	*B*	*SE*	95% CI		*β*	*R*_adj_^2^	*B*	*SE*	95% CI		*β*	*R*_adj_^2^	*B*	*SE*	95% CI		*β*	*R*_adj_^2^
			*LL*	*UL*					*LL*	*UL*					*LL*	*UL*		
Step 1						.05^***^						.05^***^						.04^***^
Constant	.67^***^	.09	.48	.85			.67^***^	.09	.48	.86			.66^***^	.09	.48	.85		
Age	−.02^***^	.00	−.02	−.01	−.22		−.02^***^	.00	−.02	−.01	−.22		−.02^***^	.00	−.02	−.01	−.21	
Step 2						.11^***^						.11^***^						.14^***^
Constant	.38^***^	.10	.19	57			.37^***^	.09	.18	.55			.29^**^	.10	.10	.46		
Age	−.01^***^	.00	−.02	−0.01	−.18		−.01^***^	.00	−.02	−.01	−.18		−.01^***^	.00	−.02	−.01	−.17	
Victimization	.05^***^	.01	.04	.07	.20		.05^***^	.01	.04	.07	.22		.07^***^	.01	.05	.08	.28	
Norm conformity	.12^***^	.03	.06	.19	.12		.10^***^	.03	.04	.16	.10		.12^***^	.03	.06	.19	.12	
Step 3						.11^***^						.11^***^						.14^***^
Constant	.38^***^	.10	.19	.56			.37^***^	.10	.18	.56			.28^**^	.10	.09	.46		
Age	−.01^***^	.00	−.02	−.01	−.18		−.01^***^	.00	−.02	−.01	−.18		−.01^***^	.00	−.02	−.01	−.17	
Victimization	.05^***^	.01	.03	.06	.19		.06^***^	.07	.04	.07	.23		.07^***^	.01	.05	.08	.27	
Norm conformity	.09^*^	.04	.01	.16	.09		.11^**^	.04	.04	.20	.11		.10^**^	.04	.03	.17	.10	
Vic × Norms	.01	.00	−.01	.02	.06		.00	.01	−.02	.01	−.02		.01	.01	−.01	.02	.04	

^*^p < .05. ^**^p < .01. ^***^p < .001

**Table 5:** Hierarchical regressions for depth of victimization

	Model 1: DV = Anxiety						Model 2: DV = Depression						Model 3: DV = PTSD					
	*B*	*SE*	95% CI		*β*	*R*_adj_^2^	*B*	*SE*	95% CI		*β*	*R*_adj_^2^	*B*	*SE*	95% CI		*β*	*R*_adj_^2^
			*LL*	*UL*					*LL*	*UL*					*LL*	*UL*		
Step 1						.05^***^						.05^***^						.04^***^
Constant	.67^***^	.09	.50	.85			.67^***^	.09	.49	.85			.66^***^	.09	.47	.83		
Age	−.02^***^	.00	−.02	−.01	−.22		−.02^***^	.00	−.02	−.01	−.22		−.02^***^	.00	−.02	−.01	−.21	
Step 2						.11^***^						.11^***^						.14^***^
Constant	.42^***^	.09	.24	.61			.41^***^	.09	.22	.59			.35^***^	.09	.18	.54		
Age	−.01^***^	.00	−.02	−.01	−.18		−.01^***^	.00	−.02	−.01	−.19		−.01^***^	.00	−.02	−.01	−.18	
Victimization	.02^***^	.00	.02	.03	.20		.03^***^	.00	.02	.03	.24		.03^***^	.00	.02	.04	.27	
Norm conformity	.12^***^	.03	.06	.19	.12		.10^***^	.03	.04	.16	.10		.12^***^	.03	.06	.19	.12	
Step 3						.11^***^						.11^***^						.14^***^
Constant	.42^***^	.09	.24	.60			.41^***^	.10	.23	.60			.35^***^	.09	.17	.53		
Age	−.01^***^	.00	−.02	−.01	−.18		−.01^***^	.00	−.02	−.01	−.19		−.01^***^	.00	−.02	−.01	−.18	
Victimization	.02^***^	.00	.02	.03	.20		.03^***^	.00	.02	.03	.24		.03^***^	.00	.02	.04	.27	
Norm conformity	.09^*^	.03	.02	.16	.09		.11^**^	.04	.05	.19	.11		.10^**^	.03	.03	.17	.10	
Vic × Norms	.00	.00	.00	.01	.05		.00	.00	−.01	.00	−.02		.00	.00	.00	.01	.04	

^*^p < .05. ^**^p < .01. ^***^p < .001

We also tested whether conformity to masculine norms moderated the effect of victimization on mental disorders. Specifically, we included an interaction between victimization and conformity to masculine norms for anxiety, depression, and PTSD in each manifestation of victimization—incidence, breadth, and depth, for a total of nine interaction effects across all models. Conformity to masculine norms did not increased model fit and all interaction effects were non-significant, indicating that conformity to masculine norms did not moderate the effect of sexual victimization on mental disorders (see Tables 3, 4, 5).

## Discussion

In a sample of 1124 British heterosexual men, we found a high rate of sexual victimization by women. Overall, 71% of participants reported experiencing some form of male sexual victimization by women at least once during their lifetime. These results were higher than those reported in the NISVS study from which the sexual victimization survey was based on (for breakdown of incidence rates, see Black et al., 2011). Perhaps a high estimate was gained due to modifications made to the survey for the present study, such as the novel inclusion of multiple forms of sexual victimization originally not measured (e.g., digital stimulation, object penetration) and the heightened anonymity of the online survey format. However, the findings of the current study are not dissimilar to other estimates in the existing literature. For example, studies on American college students report rates of male sexual victimization as high 73% (Waldner-Haugrud & Magruder, 1995), with specifically female-perpetrated victimization as high as 70% (Fiebert & Tucci, 1998). More recent studies in German and Turkish samples have reported male sexual victimization as high as 65% (Depraetere et al., 2020). The findings of the present study indicate that high levels of male sexual victimization in Britain may match those reported in other countries. Clearly, the occurrence of male sexual victimization by women is a prevalent issue that requires further attention.

In the current study, 57% of the sample was victimized more than once and 45% of the sample experienced sexual victimization more than twice. This is in line with prior literature establishing that victims have a greater risk for sexual re-victimization (Classen et al., 2005; Messman-Moore & Long, 2003). Further, some of the most common forms of sexual victimization experienced in the sample included public harassment (25%), unwanted kissing (32%), unwanted fondling (45%), forced manual stimulation when unable to consent (25%), attempted vaginal sex (36%), and attempted oral or anal sex (25%). Irrespective of tactic used, 40% of the sample experienced unwanted performance of attempted or completed vaginal or anal penetration. Further, participants reported that exploitation of inability to consent (29%) and psychological coercion (33%) were more commonly used tactics, compared to the use of physical force or threats of physical harm (5%). These findings are indicative of prior research on male sexual victimization by women. A synthesis by Depraetere et al. (2020) reported that a substantial number of studies yielded significant incidence rates of unwanted kissing, touching, oral sex, anal sex, and being made to penetrate in male victims. Further, studies indicate that female perpetrators are less likely to use physically forceful tactics compared to male perpetrators; rather, exploiting a victim’s incapacitated state or using psychological coercion (e.g., repeated requests; instigating sexual arousal) are frequently reported by female perpetrators (Depraetere et al., 2020). These studies signify the importance of using informed and gender-inclusive survey materials. Studies that exclude lesser forms of sexual victimization, such as sexual harassment, kissing, or groping, may be missing relevant incidents of sexual victimization in the male population. Further, if physical force is the only tactic considered, the majority of male sexual victimization by women may remain unexplored.

Results of the present study also support that sexual victimization is associated with anxiety, depression, and PTSD. Notably, victimization and PTSD had a particularly robust relationship in the current sample. These findings are in line with a substantial body of research showing that sexual victimization is associated with mental disorders regardless of the victim’s gender (Chen et al., 2010; Dworkin et al., 2017). The psychological suffering that male victims experience indicates the necessity of continued research on the topic of male sexual victimization.

Interestingly, results of the present study also indicated that conformity to masculine norms did not alter the psychological impact of sexual victimization. These findings fail to provide support for theories that gender norms place an additional burden on male victims due to threats to gender identity (e.g., Fisher & Pina, 2013; Weiss, 2010). Although there are few empirical studies on the influence of gender norms on the psychological suffering of male victims, two related studies on male sexual victimization supported that psychological distress and suicide attempts were positively associated with conformity to masculine norms (Easton, 2014; Easton et al., 2013). This prior work largely sampled members of sexual victimization organizations who experienced childhood sexual victimization by male clergy members. The discrepancy between the findings of the present study and of prior literature signifies the necessity for delving deeper into the topic of male sexual victimization, as well as the importance of employing consistent research methodologies to facilitate comparison.

There are many potential strengths of the present study. Compared to previous research that often uses college age American samples, the current study utilized a large British sample increasing the range of our understanding of female-perpetrated male sexual victimization. Further, a robust measure was utilized to determine the pervasiveness of male sexual victimization by women. The measure’s broad inclusion criteria of what constitutes sexual victimization within the current study may have made a substantial difference in participant reporting (see Depraetere et al., 2020). Many institutions use gender-biased definitions that often exclude less severe and male-specific forms of sexual victimization (Stemple & Meyer, 2014). Surveys may exclude noncoital victimization, in which acts such as sexual harassment, kissing, or touching are not measured. Further, most studies operationally define rape by the penetration of the victim but exclude being made to penetrate (Stemple & Meyer, 2014). These methodological choices lead to the underrepresentation of male victimization in incidence estimates (Anderson et al., 2020). Further, studies on sexual victimization measurement support that behaviorally specific questions generate higher estimates of victimization (e.g., Fisher et al., 2000; Koss, 1993b). For example, the use of terms (e.g., “rape”) without operationalized definitions (e.g., “being penetrated or being made to penetrate”) leads to less disclosure (World Health Organization, 2013), particularly because many victims do not perceive their own experiences with unwanted sexual activity as sexual victimization (Muehlenhard et al., 1992; Peterson et al., 2011). Therefore, the use of behaviorally specific questions may have positively impacted reporting of sexual victimization in the current study.

There were several limitations to the study. The sexual victimization survey may have garnered a higher incidence estimate if the syntax of the questions was presented differently. Research on victimization measurement indicates that the tactic of sexual victimization (e.g., use of physical force, drugging) should be presented first, and followed by the type of sex act in order to engage participant memory more effectively (Abbey et al., 2005). Further, although we examine the moderation of gender norms on mental disorders, the present study did not provide insights into the influence of gender norms on other aspects of male sexual victimization, such as hesitancy of reporting. In an exploration of victims’ narratives by Weiss (2010), male victims expressed shame for being unable to protect themselves and self-blame for failing in their masculine role. For fear of humiliation, male victims may face difficulty acknowledging to themselves that their experiences of unwanted sexual activity are forms of sexual victimization. These threats to masculine identity may impact the likelihood of recognizing and reporting incidents of sexual victimization (Davies, 2002).

Finally, the correlational nature of the current work clearly precludes strong causal claims. Our analytic approach focused on the association between sexual victimization and subsequent mental disorders. The causal direction from victimization to mental disorders is established in the literature (e.g., Krahé & Berger, 2017); thus, our analysis is consistent with the prevailing understanding that sexual victimization precipitates poor mental health. However, there is also evidence that individuals experiencing mental disorders may be especially vulnerable to sexual victimization. Previous studies have shown that women and men who are experiencing mental disorders are more likely to be sexually victimized (Miles et al., 2022; Vik et al., 2019). Moreover, a study on student populations found that depression can be both an outcome and a predictor of sexual victimization (Krahé & Berger, 2017). This raises the prospect that mental disorders can be an important factor in re-victimization. Our findings might point in the opposite casual direction, from vulnerability to victimization. Cross-sectional studies cannot disentangle causality, and future work should further examine the relationship between victimization and mental disorders longitudinally.

In conclusion, the current study further illuminates the occurrence of male sexual victimization by women and counters cultural myths prescribing that men cannot experience psychological suffering as a result of sexual victimization. The findings of the present study support that sexual victimization is a prevalent issue that may impact a significant percentage of the male population. Further, the study supports that male sexual victimization is of particular importance due to the association between victimization and experiencing mental disorders, namely anxiety, depression, and PTSD. Conformity to masculine norms did not moderate the relationship between mental disorders and sexual victimization in this study. Future psychological research should utilize consistent methodologies and gender-inclusive measures. By resolving discrepancies in the literature, researchers may be better positioned to understand the issue and to provide aid to those who experience male sexual victimization by women.

## Data Availability

Data and materials are available at https://osf.io/d672x/

## Code Availability

NA.
